# Frontotemporal Dementias in Latin America: History, Epidemiology, Genetics, and Clinical Research

**DOI:** 10.3389/fneur.2021.710332

**Published:** 2021-09-06

**Authors:** Jorge J. Llibre-Guerra, Maria Isabel Behrens, Mirna Lie Hosogi, Lucia Montero, Teresa Torralva, Nilton Custodio, Erika Mariana Longoria-Ibarrola, Margarita Giraldo-Chica, David Aguillón, Angela Hardi, Gladys E. Maestre, Valeria Contreras, Celeste Doldan, Lissette Duque-Peñailillo, Heike Hesse, Norbel Roman, Dhara Angelina Santana-Trinidad, Christian Schenk, Ninoska Ocampo-Barba, Ricardo López-Contreras, Ricardo Nitrini

**Affiliations:** ^1^Department of Neurology, Washington University School of Medicine, St. Louis, MO, United States; ^2^Departamento de Neurología y Neurocirugía Hospital Clínico Universidad de Chile, Departamento de Neurociencia, Centro de Investigación Clínica Avanzada (CICA), Facultad de Medicina, Universidad de Chile, Santiago de Chile, Chile; ^3^Departamento de Psiquiatría y Neurología, Clínica Alemana de Santiago, Universidad del Desarrollo, Santiago, Chile; ^4^Departmento de Neurologia, Faculdade de Medicina da Universidade de São Paulo, São Paulo, Brazil; ^5^Laboratory of Neuropsychology (LNPS), Institute of Cognitive and Translational Neuroscience (INCYT), INECO Foundation, Favaloro University, Buenos Aires, Argentina; ^6^Unidad de Diagnóstico de Deterioro Cognitivo y Prevención de Demencia, Instituto Peruano de Neurociencias, Lima, Peru; ^7^Instituto Nacional de Neurologia y Neurocirugia MVS, Universidad Nacional Autonoma de Mexico, Mexico City, Mexico; ^8^Grupo de Neurociencias de Antioquia, Facultad de Medicina, Universidad de Antioquia, Medellín, Colombia; ^9^Becker Medical Library, Washington University School of Medicine, St. Louis, MO, United States; ^10^Departament of Neurosciences and Alzheimer's Disease Resource Center for Minority Aging Research, University of Texas Rio Grande Valley, Brownsville, TX, United States; ^11^Departamento de Neuropsicología, Hospital de Clínicas Dr Manuel Quintela, Universidad de la República, Montevideo, Uruguay; ^12^Departamento de Neuropsicología Cognitiva, Clínica Especializada en Neurociencias Física y Cognitiva CEFYC, Asunción, Paraguay; ^13^Cognitive Disorders Unit, Neuromedicenter, Quito, Ecuador; ^14^Observatorio COVID-19, Universidad Tecnológica Centroamericana, Tegucigalpa, Honduras; ^15^Hospital Social Security of Costa Rica, Universidad de Costa Rica, San Jose, Costa Rica; ^16^Hospital Traumatológico Ney Arias, Santo Domingo, Dominican Republic; ^17^Sección de Neurología, Dept. de Medicina. Recinto de Ciencias Médicas- Universidad de Puerto Rico, San Juan, Puerto Rico; ^18^Instituto Boliviano de Neurociencia Cognitiva, Universidad Autónoma Gabriel René Moreno, Santa Cruz de la Sierra, Bolivia; ^19^Clínica de Memoria, Servicio de Neurología, Instituto Salvadoreño del Seguro Social, San Salvador, El Salvador

**Keywords:** frontotemporal dementia, Latin America, history, prevalence, genetics, biomarkers

## Abstract

**Introduction:** The historical development, frequency, and impact of frontotemporal dementia (FTD) are less clear in Latin America than in high-income countries. Although there is a growing number of dementia studies in Latin America, little is known collectively about FTD prevalence studies by country, clinical heterogeneity, risk factors, and genetics in Latin American countries.

**Methods:** A systematic review was completed, aimed at identifying the frequency, clinical heterogeneity, and genetics studies of FTD in Latin American populations. The search strategies used a combination of standardized terms for FTD and related disorders. In addition, at least one author per Latin American country summarized the available literature. Collaborative or regional studies were reviewed during consensus meetings.

**Results:** The first FTD reports published in Latin America were mostly case reports. The last two decades marked a substantial increase in the number of FTD research in Latin American countries. Brazil (165), Argentina (84), Colombia (26), and Chile (23) are the countries with the larger numbers of FTD published studies. Most of the research has focused on clinical and neuropsychological features (*n* = 247), including the local adaptation of neuropsychological and behavioral assessment batteries. However, there are little to no large studies on prevalence (*n* = 4), biomarkers (*n* = 9), or neuropathology (*n* = 3) of FTD.

**Conclusions:** Future FTD studies will be required in Latin America, albeit with a greater emphasis on clinical diagnosis, genetics, biomarkers, and neuropathological studies. Regional and country-level efforts should seek better estimations of the prevalence, incidence, and economic impact of FTD syndromes.

## Introduction

Frontotemporal lobar degeneration (FTLD) is a neuropathological designation used to identify a group of neurodegenerative diseases of the frontal and anterior temporal lobes, typically associated with specific pathologies (1). In most cases, FTLD features pathological inclusions of either the microtubule-associated protein tau (MAPT) or the transactive response DNA-binding protein of 43 kDa (TDP-43), named FTLD-tau and FTLD-TDP, respectively (2). TDP-43 is the major pathological protein deposited in FTLD and amyotrophic lateral sclerosis (ALS) (3–5). FTLD can be sporadic or hereditary, the latter most commonly due to mutations in several genes, such as MAPT, progranulin (GRN), TARDBP, or chromosome 9 open reading frame 72 (C9orf72) expansion.

The core clinical syndromes associated with FTLD are behavioral or language symptoms and are generally called frontotemporal dementia (FTD). There are three main clinical variants distinguished by early and predominant symptoms: behavior variant frontotemporal dementia (bvFTD); semantic variant primary progressive aphasia (svPPA); and non-fluent variant primary progressive aphasia (nfvPPA) (6) bvFTD accounts for roughly 60% of FTD cases, and the other 40% are language variants of FTD (7). Related FTD disorders include frontotemporal dementia with motor neuron disease (FTD-MND), progressive supranuclear palsy syndrome (PSP-S), and corticobasal syndrome (CBS).

FTD is the second most common dementia disorder in individuals under the age of 65 years old and accounts for 5–10% of dementia patients older than 65 years (3, 4). In the US, the total number of cases with FTD syndromes range from 15 to 22 per 100,000 people in the US (8, 9) with ~20,000 to 30,000 persons living with FTD (9). The incidence of FTD is estimated to be 1.61 to 4.1 cases per 100,000 people annually (8, 9).

FTD is likely underdiagnosed due to the relatively low recognition within the medical community, little disease awareness in the population, and the overlap with a multitude of psychiatric disorders (10–13). Therefore, prevalence studies on bvFTD and the other FTD syndromes are challenging because many cases are misclassified, as the disease is largely unrecognized (7, 9).

The frequency and correlates of the impact of FTD are less clear in Latin American countries. Although there is a growing number of dementia studies in Latin America, little is known collectively about FTD studies by country, its clinical heterogeneity, risk factors, and genetics in Latin American countries. Therefore, we aimed to systematically review FTD studies reported in Latin America. This systematic review offers an overview of the history and evolution of FTD in Latin America and reports on FTD prevalence and clinical and neuropsychological syndromes. This is followed by a review of the biomarkers, neuropathology, and genetic studies in the region.

## Methods

A systematic review was completed at identifying and describing the frequency, clinical heterogeneity, and research studies on FTDs in Latin American populations. The search strategy was developed with assistance from a research committee formed by a medical librarian, representatives from multiple Latin American countries (local dementia experts and clinical researchers), and other stakeholders with expertise in FTD. The research committee provided feedback and guidance on the proposed search strategies, selection criteria, and data analysis approach.

The published literature was searched using strategies designed by a medical librarian for the concepts of FTD, Latin American countries, and related synonyms. These strategies were created using a combination of controlled vocabulary terms and keywords and were executed in Medline (Ovid) 1946-, Embase.com 1947-, Scopus 1823-, PsycInfo, Cochrane Library (including CENTRAL), LILACS 1982-, and SciElo.org. No filters or limits were applied to the search. All searches were completed on September 14, 2020. Full search strategies are provided in the Supplementary Material. A total of 483 results were retrieved from the literature search and imported into Endnote. Dementia experts and clinical researchers from Latin America (at least one per country) were asked to provide information on FTD publications in the Latin American region, yielding 213 records through hand-searching. A total of 696 citations retrieved by these methods (literature search + dementia experts reports) were compiled and screened for duplicates. Duplicate citations (*n* = 272) were accurately identified and removed for a total of 424 unique citations.

**Figure 1:**
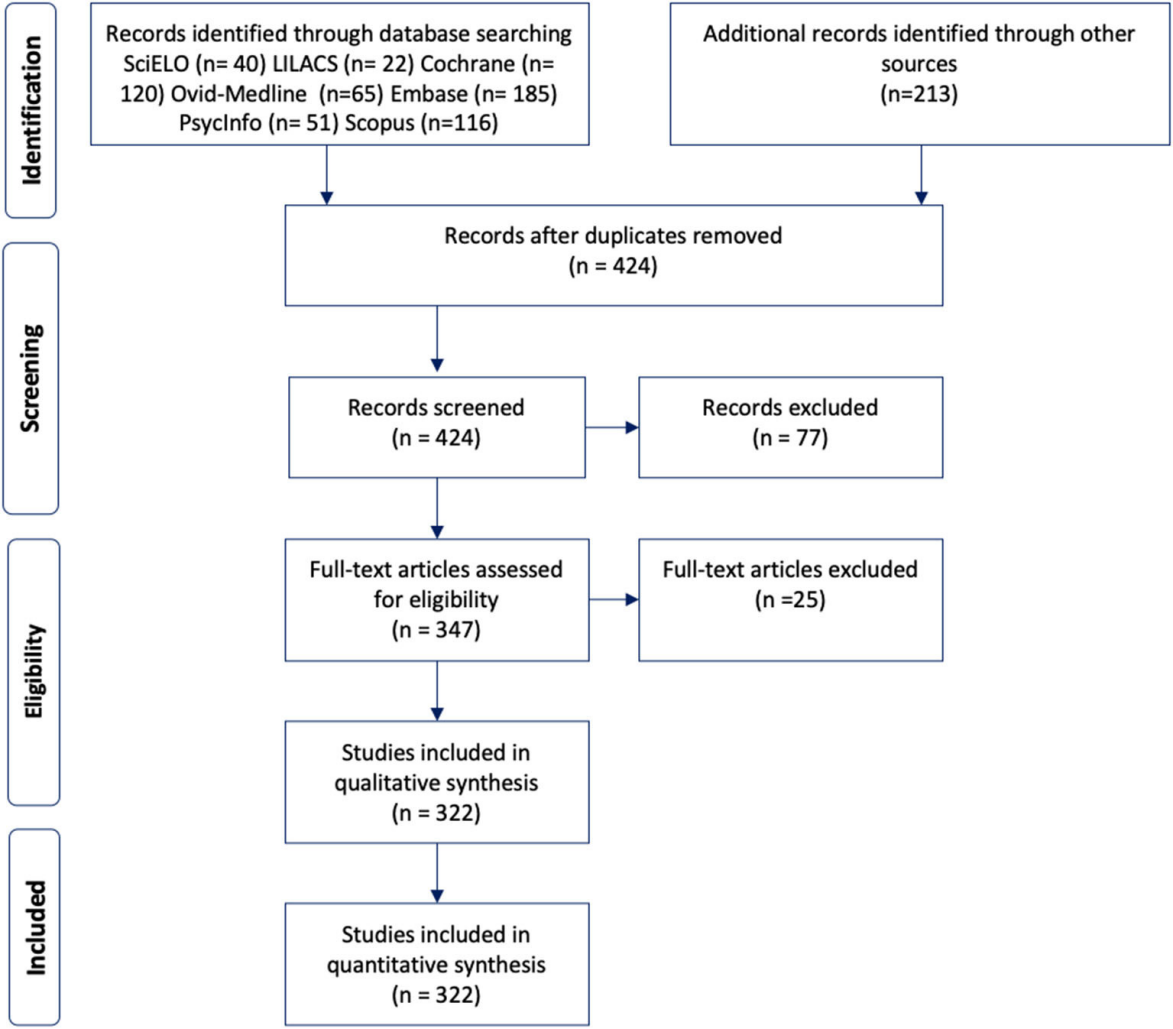
Diagram of the study selection process for the systematic review.

After removing duplicates all citations (*n* = 424) were screened for appropriateness against the inclusion and exclusion criteria. Studies were included if they reported on (1) clinical features of FTD and (2) reports from populations living in Latin American countries. Reports describing non-FTD studies were excluded from this study. Studies published by Latin American authors but that did not include Latin American participants, as well as studies of Hispanics not living in Latin American countries, were also excluded. Studies that were done in collaboration (regional or international) were included if they involved Latin American participants. Poster presentations and meetings abstracts were excluded, except in areas where its relevance was sought to contribute to the understanding of FTD in Latin America (e.g., genetics and prevalence studies). After the abstract screening phase, studies that met the inclusion criteria (*n* = 398) underwent full-text assessment for eligibility (second screening stage) and were selected based on their relevance. Three hundred and twenty-two (322) peer-reviewed publications were selected for the final analysis (Figure 1).

At least one author per Latin American country summarized the FTD literature that was found in their country; collaborative or regional studies were reviewed during consensus meetings. From each research study, information on sociodemographic characteristics, country report, and genetics were extracted. Information on clinical features (age at onset, age of death, disease duration, clinical presentation, atypical manifestations, and neurological findings) were obtained when available. We considered each symptom or sign as present or absent when clearly stated in the reports. A group composed of three of the authors (MIB, JL, and RN) received all the comments and classified the FTD reports from Latin American countries according to publication date (before 2000 or after 2000), epidemiology, clinical presentation, genetics, and neuropathology.

This study was reported according to the Preferred Reporting Items for Systematic Review and Meta-Analysis (PRISMA) guidelines (14).

## Results

**Figure 2:**
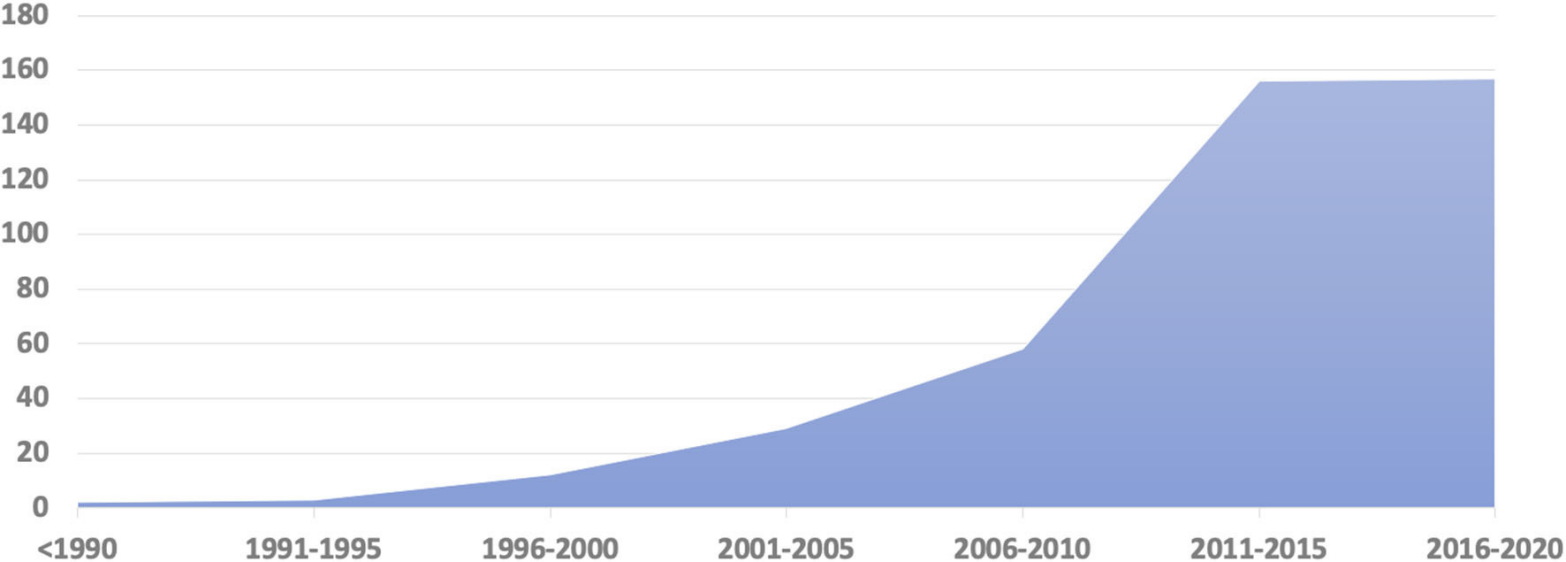
Frontotemporal dementia and related disorders publications in Latin America by years.

**Figure 3:**
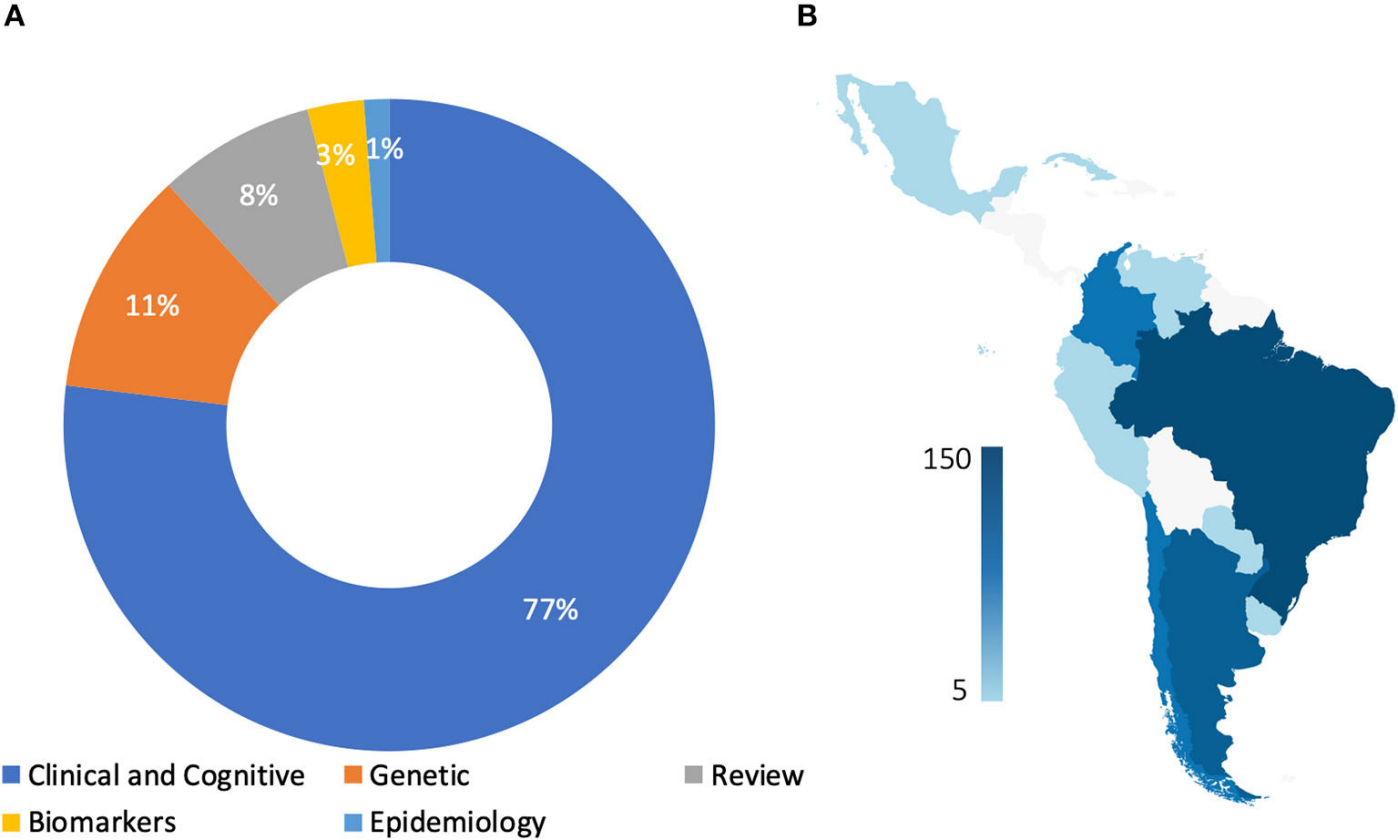
Frontotemporal dementia and related disorders research in Latin America by country and topics. Panel **(A)** describe percentage of frontotemporal dementia studies in Latin America by research area. Panel **(B)**, describe the number of FTD publications by country.

A total of 322 peer-reviewed publications were included in the final review and analysis. Twenty-two peer-reviewed papers were published during the twentieth century and provide an overview of the history and early development of the FTD field in Latin America. The early 2000s marked an increase in the research related to FTD, with a spike between 2010 and 2015 (Figure 2). Brazil (165), Argentina (84), Colombia (26), and Chile (23) are the countries with the larger number of published reports (Table 1). Most of the research has focused on clinical and neuropsychological features (*n* = 247), including the local adaptation of neuropsychological and behavioral assessment batteries. However, there are little to no large studies on prevalence (*n* = 4), biomarkers (*n* = 9), or genetics (*n* = 36) of FTD (Figure 3).

**Table 1:** Frontotemporal dementia and related disorders publications in Latin American countries.

**Country**	**Clinical cognitive**				Genetics^**^	**Biomarkers**	**Reviews by Latin America authors**	**Total**
	bvFTD^*^	**FTD-ALS**	**PPA**	**CBS/PSPs**				
Brazil	74	8	33	2	25	5	15	165
Argentina	54	–	6	9	3	3	2	77
Colombia	16	–	5	–	1	1	3	26
Chile	11	1	3	3	1	–	4	23
Peru	8	1	1	1	–	–	–	11
Uruguay	2	–	1	–	1	–	–	4
Mexico	2	–	1	–	2	–	–	5
Cuba	1	–	1	–	2	–	–	4
Venezuela	3	–	–	–	–	–	1	4
Ecuador	–	1	–	–	–	–	–	1
Dominican republic	–	–	–	–	1	–	–	1
Puerto rico	–	–	–	–	0	–	–	0
Guadeloupe	1	–	–	–	–	–	–	1
**Total**	172	11	51	15	36	9	25	322

*FTD, frontotemporal dementia; bvFTD, behavioral variant FTD; ALS, amyotrophic lateral sclerosis CBS/PSPS, corticobasal syndrome/progressive supranuclear palsy syndrome; PPA, primary progressive aphasia*.

* *2 additional epidemiological abstracts are discussed but not included in this table*.

** *17 additional abstracts are discussed but not included in this table (eight from Brazil, five from Argentina, one from Colombia, one from Cuba, one from the Dominican Republic, and one from Puerto Rico). There are 22 regional collaborative studies (nine between Argentina, Chile, and Colombia; seven between Argentina and Colombia; two between Argentina and Chile; two between Argentina and Perú; one between Brazil and Chile; and one between Cuba, Uruguay, and Ireland. Collaborative studies were assigned to the country of the first author or to the nationality of the patients included*.

### Evolution of FTD in Latin America (Twentieth Century)

The first Latin America publication of bvFTD associated with ALS was reported by Tretiakoff and Amorim in 1924 (15). The case report described a young woman with absolute indifference, complete absence of affective feelings, and severe impairment of memory, which were followed by motor neuron signs of ALS. The neuropathological examination of the case described evidence of ALS but no signs of other dementia-causing pathology in the brain. The authors hypothesized that dementia was part of ALS and recommended the search for signs of involvement of motor neurons in dementia cases, a practice currently accepted in the clinical workup of FTD cases.

In 1987, Nitrini et al. described three patients with progressive supranuclear palsy (PSP) in Brazil who presented with elementary motor perseveration before the appearance of any other distinctive features of the disease (16). The authors suggested that motor perseveration was an important sign for early diagnosis and a key element for the clinical characterization of PSP.

In 1989, Oliveira et al. reported a patient with difficulties in comprehension of written texts that were followed by other language disturbances and dementia (17).

In 1992, Trevisol-Bittencourt, also from Brazil, reported a case of PSP with dementia and highlighted diagnostics challenges due to the presence of both “subcortical dementia” and frontal lobe syndrome (18).

In 1994, Donoso et al., from Chile, reported six cases of degenerative dementia with frontal or frontotemporal hypoperfusion on SPECT (19). Five cases were classified as “frontal progressive dementia,” whereas one patient had progressive aphasia. In the same year, Leiguarda et al., from Argentina, in collaboration with the Institute of Neurology of the University College of London, published a description on apraxia and corticobasal degeneration, followed by a relevant contribution to the knowledge of apraxia (20–23).

Several case descriptions populated the regional literature from 1995 to the 2000s.

In a publication on the diagnosis of 100 patients evaluated in an outpatient memory clinic in Brazil, Nitrini et al. (24), reported two cases classified as frontal lobe dementia. In Delgado et al. (25) from Brazil, reported a non-fluent PPA with MRI revealing atrophy on the left perisylvian fissure region. In 1998, three patients with neuropathologically confirmed FTD with motor neuron disease who manifested hallucinations were reported, and a hypothesis about the occurrence of hallucination in dementia associated with MND was proposed by Nitrini and Rosemberg (26). Caixeta and Nitrini described the clinical features of 10 Brazilian patients with FTD, searching for qualitative and quantitative behavioral changes. Disinhibition predominated in six patients, apathy in four, while all patients manifested repetitive behaviors (27).

In 1998 Allegri et al. (28) compared the cognitive profile of 12 Argentinian patients with bvFTD and 20 patients with probable Alzheimer's disease, showing that FTD patients scored significantly better than AD patients in memory tests, calculations, visuospatial abilities, and the naming test. AD patients performed better on executive tasks.

A clinical and pathological report of a case of FTD associated with ALS was published by De Brito-Marques and De Mello (29), describing neuropathological findings similar to those described by Gustafson (30). In Doval and Gaviria (31), from Venezuela, published a review on FTD emphasizing their opinion that FTD was not a new clinical entity but a redefinition of the classical Pick's disease, an opinion that reflected the central concept on dementia diagnosis during most of the twentieth century in Latin America and most of the Western countries (32, 33).

Finally, a Chilean and an Uruguayan investigator participated in the development of the Frontal Assessment Battery (FAB) test (34). After these early papers, the number of scientific publications increased exponentially (Figure 2).

### Clinical Presentation and Neuropsychology of FTD in Latin America

In the decade between 2000 and 2010, most of the publications described clinical, neuropsychological features, and structural imaging of FTD cases (Table 1). In addition, several authors have raised concerns about the difficulties and under-diagnosis of FTD and related disorders in Latin American countries (35–38).

#### FTD Prevalence Estimates in Latin America

There are few studies on the prevalence of FTD in Latin American countries. In a systematic review, Custodio et al. (39) described FTD prevalence in three Latin American countries [Venezuela (40), Perú (41), Brazil (42, 43)] ranging from 1.2 to 1.7 per 1,000. In a population-based study in an area of Maracaibo, Venezuela, in subjects older than 54 years, the prevalence of all-cause dementia was 8.04%, while the prevalence of FTD was 1.5% (44). There are also two studies presented at International conferences: one population study from Mexico with 2003 participants estimated a prevalence of FTD of 0.9%, and another 5-year population study with nearly 3,000 participants from Habana Cuba found a prevalence of FTD of 1.1% (45).

Other studies report the frequency of FTD within dementia cohorts in memory clinics. One study in Brazil reported a 3.5% frequency of FTD in 261 dementia cases assessed between 1989 and 1998, using the Brun criteria (46). Two studies from Memory clinics in Colombia report an FTD frequency between 11.5 and 12.9% (47, 48). Finally, one study in a memory clinic in Santiago, Chile, found 57 FTD patients among 3,700 dementia patients assessed between 1981 and 2008, using the Neary et al. (3) criteria in a memory clinic in Santiago (1.5%) (49).

#### FTD Clinical and Neuropsychology Studies in Latin America

The majority of the publications in Latin America (*n* = 247) describe the clinical features of FTD. Brazil has the largest number of publications on the clinical and neuropsychological characteristics of FTD. Also, there are case reports of late-onset (>85) bvFTD (50). It is also interesting to mention a paper on long-term severe mental disorders preceding bvFTD in a Brazilian cohort (51).

Argentina has several papers on the brain structural correlates of executive and social cognition and also decision-making cognition and moral judgment in bvFTD and PPA (52–67).

The relationship between FTD and creativity and theory of mind has also been explored (68–70). Recent papers also report the use of automated computational approaches and machine learning to aid in the diagnosis of FTD (71, 72). Taragano et al. (73–75) published several papers on mild behavioral impairment and Tabernero et al. (76, 77) published papers on facial emotion recognition.

There are several publications related to the validation of tests in Spanish and Brazilian Portuguese (78–85). It is also important to mention that a group at the Institute of Cognitive Neurology (INECO) in Argentina developed the INECO Frontal Screening (IFS) as a brief, sensitive, and specific tool to assess executive functions in dementia (86). This test has also been validated in Chile (83), Perú (87), and Brazil (88).

Finally, there are many publications of collaborative research and between Latin-American and the US or European Countries. It is interesting to note that there are also many joint publications within the region, namely, Argentina–Perú (79, 87), Argentina–Colombia–Chile (56, 66, 89, 90), Argentina–Chile (67), and Argentina–Colombia (54, 63, 72).

#### Genetics of FTD in Latin America

**Figure 4:**
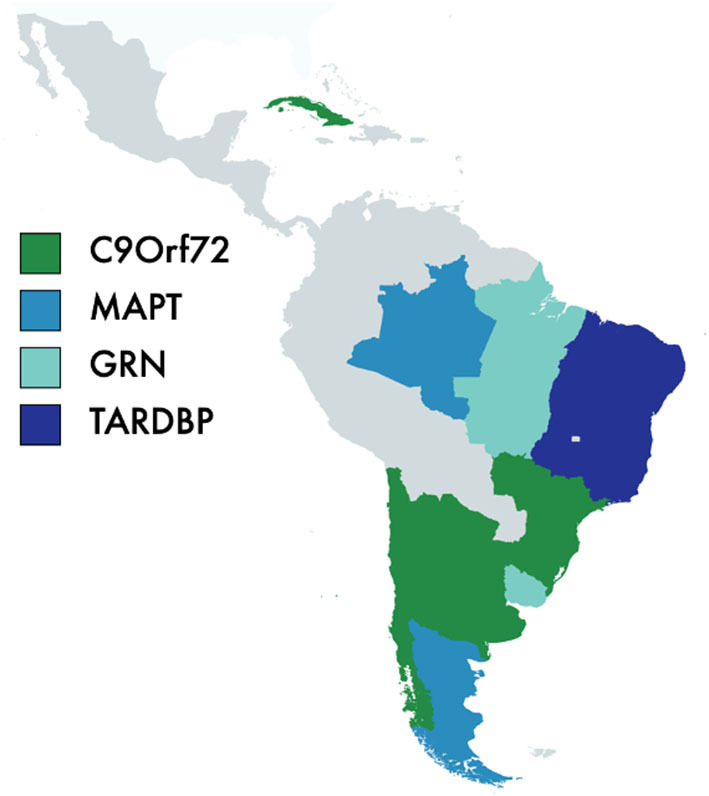
Frontotemporal dementia and genetic in Latin America. C9orf72, chromosome 9 open reading frame 72; MAPT, Microtubule-Associated Protein Tau; GRN, Progranulin; TARDBP, 43-kDa transactive response (TAR)-DNA-binding protein.

The genetics of FTD syndromes in Latin America remains understudied, with no FTD large genetic studies aimed at identifying novel or functional rare variants in the region. However, there are family reports from various countries, including Brazil (91, 92), Argentina (93), Uruguay (94), Cuba (95), Chile (96), and Caribbean origin families (97) (Figure 4). Families carrying C9ORF72 have been described in Chile (96), Cuba (95), Brazil (98, 99), and Argentina (100, 101), presenting with a significant phenotypic heterogeneity (ALS vs. bvFTD vs. bvFTD-MND). Families featuring GRN pathogenic variants have been described in Brazil (91, 92), Uruguay (94), Argentina (102), and the Caribbean (97). MAPT mutations have only been reported in Brazilian (103), and Argentinian (104), families, while TARDBP mutations have only been reported in Brazil. A missense mutation (R93C) in the valosin-containing protein (gene) was also described in a Brazilian family presenting with progressive myopathy together with clinical and cognitive features of FTD (105). The study of other genetic factors related to FTD is also limited in Latin America (95, 106). Recent findings by Nascimento et al. showed a higher frequency of TDP-43 pathology in cognitively healthy Asian Americans compared to Caucasians living in Brazil (107); similarly, Hardiman et al. (95) described a higher frequency of C9orf72 repeat expansions in an Irish FTD-ALS cohort compared to a similar cohort in Cuba, suggesting possible differences in FTD-related neuropathology and neurodegeneration according to ethnicity. Future studies should address whether observed differences are explained by health and social disparities or possible ethnic-related protective factors against clinical expression of TDP-43 proteinopathies.

#### FTD Biomarkers and Neuropathology in Latin America

We found relatively few reports with extensive documentation on neuropathology, biomarker profiles, and disease progression in Latin American populations, making genotype–phenotype correlations difficult in the region. Although the use of dementia biomarkers is not widespread across Latin American countries, studies using biomarkers in FTD cohorts are available in Argentina (108), Brazil (109), and Uruguay (110). Neuroimaging studies in Latin American populations mainly describe structural findings consistent with the atrophy patterns reported in FTD studies from high-income countries. Neuropathological reports were scarce and only available in Brazilian cohorts (107, 111, 112).

### Primary Progressive Aphasia

In our review, we found a relatively low number of PPA reports in Latin America, with two reports before 2,000, 13 between 2000 and 2010, and 42 from 2011 to 2020. Similar to the findings in bvFTD, Brazil has the greatest number of publications in Latin America (36 vs. 63, respectively). There are PPA studies reported in Argentina (*n* = 12), Chile (*n* = 1), Colombia (*n* = 4), Peru (*n* = 2), Cuba (*n* = 1), Mexico (*n* = 1), and three collaborative studies: Argentina/Chile/Australia (*n* = 1), Argentina/Chile/Colombia, and Australia (*n* = 2). Some of these manuscripts have already been cited in the previous sections.

According to the available reports, the frequency of PPA syndromes is low. Diagnostic classification also varies within PPA cohorts and country reports. In a Chilean study by Donoso et al. (49), 15.8% of the cases in an FTD cohort received a PPA diagnosis. In a consecutive series of 100 Brazilian PPA cases (113) using the classification proposed by Gorno-Tempini et al. (114), 35 were diagnosed as svPPA, 29 as nfvPPA, 16 as lvPPA, and 20 were considered unclassifiable. More recently, Campanha et al. (115) described clinical characteristics of 19 featuring PPA syndrome; of those, 10 fulfilled criteria for svPPA, five for nfvPPA, three for lvPPA, and one case was considered unclassifiable. Other key features reported in the PPA have been described in Brazilian and Argentinian cohorts. In 2016, Marin et al. published a study of swallowing problems in 16 PPA patients (116). Clinical presentations as “psychiatric disorders” have also been reported (117).

Hosogi Senaha et al. (118) published the case study of a SD patient without surface dyslexia, a sign usually found in most of the SD cases to date. Similarly, in 2012, Wilson and Martínez-Cuitiño (119) reported a Spanish-speaking SD case similar to the Brazilian case. Both studies raise awareness about the possible absence of surface dyslexia in Spanish and Portuguese speakers presenting with SD, probably related to the relatively transparent orthographies of both languages. It is worth noting that both patients were able to read non-words, regular and irregular words, and foreign words correctly but with difficulties in written comprehension. In both studies, the authors associated patients' performance—reading of irregular and foreign words without meaning—with the use of the direct lexical reading process.

To the best of our knowledge, there are no large neuropathology reports on PPA cohorts. Most of the reports are based on case experiences. de Brito-Marques et al. (2011) reported a nfvPPA longitudinal case study with histopathologic analysis (120).

Strategies for languages rehabilitation in PPA has been reported from single or multiple case studies in Brazil and Mexico (121–124).

### FTD and Motor Neuron Disease

Frontotemporal dementia and motor neuron disease (FTD-MND) has been recognized as overlapping multisystem disorders (125). In this section, we focus our review on Latin American studies describing the overlap between the two conditions. Studies describing amyotrophic lateral sclerosis (ALS) cohorts without assessments of cognitive measures were excluded from this review. As mentioned above, reports of cases combining the clinical picture of MND with mental symptoms, personality change, or dementia in Latin America date back to 1924 (15).

Most of the reports on FTD/MND in Latin America are case reports, including a wide range of cognitive presentations combined with different MND syndromes, including ALS (29, 126–128) and primary lateral sclerosis (PLS) (129). There is a relative lack of large studies describing the overlap between the two conditions in Latin America, which might be related to the scarcity of adequate cognitive screening methods suitable for Spanish- and Portuguese-speaking populations with low education. To the best of our knowledge, there are only two cohorts studies exploring cognitive and behavioral presentations overlapping with MND/ALS (130, 131).

Recent efforts in the region, especially in Brazil, are on the way aimed to validate and implement adequate and more systematic cognitive screening methods in Dementia/ALS cohorts. Branco et al. (81) validated the Amyotrophic Lateral Sclerosis Cognitive Behavioral Screen (ALS-CBS) in Brazil and this is now amiable in Portuguese. A Spanish version (132) of this instrument and education adjusted measures (133) are also available and can now be used across the region.

## Discussion

The first publications of Latin American authors in the twentieth century were mostly case reports or small series of patients in which the clinical features were described. There were also a few papers with deeper reasoning on apraxia in several movement disorders and on frontal type of disinhibition in PSP. In the last two decades, most of the papers report on clinical and neuropsychological features of FTLD. Case descriptions, translations, and adaptations of neuropsychological and behavioral tests were the predominant publications by Latin American authors. Argentina has contributed with several interesting publications on social cognition and decision making.

Although there were only a few reports on FTD prevalence in the region, the reported prevalence is relatively low compared to North America and Europe. Nevertheless, future studies will be needed to determine whether this is true or a reflection that the disease is still underrecognized in Latin American counties. Available data from surveys suggest that FLD is not recognized by families and general physicians (35–38).

There are fewer studies published in Latin America related to the language variants of FTLD in comparison to the number of studies related to the bvFTD. Studies on PPA have increased substantially during recent years and also advanced from case reports to case series and, more recently, to rehabilitation initiatives. However, more sensitive methods to detect language variants are needed, especially as the classical testing methods used for English speakers cannot apply to Spanish or Portuguese speakers.

Similarly, there is a relative lack of large studies describing the overlap between FTD/MND in Latin America or exploring the cognitive and behavioral manifestations in MND/ALS, which may be related to the scarcity of adequate cognitive screening methods suitable for Spanish- and Portuguese-speaking populations with low education. Two instruments, that provide adequate cognitive screening methods suitable for Spanish and Portuguese-speaking populations with low education, have been recently validated and are expected to improve studies in this area.

Only a few neuropathological studies on FTLD have been published, and all of them are from Brazil. The relatively low number of neuropathology studies might be related to lack of resources; brain donation protocols require the existence of brain banks and trained personnel, which are scarce in the region.

Overall, most of the FTD studies are concentrated in a few countries (Brazil, Argentina, Colombia, and Chile), with only a few collaborative studies between Latin American countries and between Latin American countries and more developed centers in North America and Europe. Collaboration may represent an alternative to achieve better results and more robust studies in a region where research resources and funding are scarce.

Genetics is another area where future studies will be required. Much of the population of Latin American countries is a mixture of native American, European, African, and some Asian immigration. Therefore, it is expected to find similar mutations to those already described in the literature. In addition, the existence of novel mutations in the native American populations and the effect of admixture in gene expression, disease onset, and clinical heterogeneity should be further studied.

This systematic review also found several relevant conference abstracts with large series of cases but, unfortunately, they did not end up in peer-reviewed publications. This may be explained by a lack of privileged time and grants to perform research in Latin American countries, as well as difficulties in reaching publications in a foreign language. Although there has been improvement in the last few years, academic and governmental institutions in Latin America should implement protected time for their researchers aimed to facilitate research dissemination. Public and private funds should be directed toward research grants that will improve the research and consistency of reports coming from Latin American researchers.

## Conclusions

The analysis of the history of FTLD research in Latin America shows that there are several gaps in knowledge that remain to be explored and activities to be developed by the community. Based on our findings, we believe research on epidemiology and genetics of FTD in Latin America should be priorities. Several studies show that general physicians, neurologists, psychiatrists, and the lay public are unaware of these diseases. More collaborative studies are needed, both between Latin American countries and with developed centers in HIC, mainly on genetics and biomarkers. The interchange of undergraduate, graduate, and post-graduate students and academic professors between research centers in Latin America with those in the developed world has already started, and this is likely to change the history of FTD in Latin America. The recent formation of the Latin America network (RedLat) to study FTLD is tasked to increase these collaborations.

## Data Availability Statement

The original contributions presented in the study are included in the article/Supplementary Material, further inquiries can be directed to the corresponding author/s.

## Author Contributions

JL-G, MB, and RN: study concept and design, acquisition, analysis, interpretation of data, and drafting of the manuscript. JL-G: project administration. RN: study supervision. All authors critical revision of the manuscript for important intellectual content, had full access to all the data in the study, and take responsibility for the integrity of the data and the accuracy of the data analysis.

## Funding

MB has received funding from FONDECYT: 1190958, RN has received funds from CNPq.

## Conflict of Interest

The authors declare that the research was conducted in the absence of any commercial or financial relationships that could be construed as a potential conflict of interest.

## Publisher's Note

All claims expressed in this article are solely those of the authors and do not necessarily represent those of their affiliated organizations, or those of the publisher, the editors and the reviewers. Any product that may be evaluated in this article, or claim that may be made by its manufacturer, is not guaranteed or endorsed by the publisher.
