# Supplementary material for: Frontotemporal Dementias in Latin America: History, Epidemiology, Genetics, and Clinical Research
Source: Front Neurol. 2021 Sep 6;12:710332. doi: 10.3389/fneur.2021.710332 (PMC8450529; doi:10.3389/fneur.2021.710332)
Supplement: Supplementary file 1 [file Data_Sheet_1.docx]

**Supplemental Material:**

**Complete Search Strategies:**

**Embase.com**

=185 results on 9/14/2020

('frontotemporal dementia'/exp OR ‘frontotemporal dementia’ OR ‘frontotemporal lobe dementia’ OR ‘frontotemporal lobe dementia’ OR ‘frontal dementia’ OR ‘frontal lobe dementia’ OR ‘Wilhelmsen Lynch disease’ OR ‘frontotemporal lobar degeneration’ OR ‘disinhibition dementia Parkinsonism amyotrophy complex’ OR ‘familial Picks disease’ OR ‘Pick complex’ OR ‘Picks complex’ OR ‘hereditary dysphasic disinhibition dementia’ OR ‘FTDP-17’ OR ‘DDPAC’ OR ‘FTD-GRN’ OR ‘FTD-PGRN’ OR ‘FTLD-17 GRN’ OR ‘FTLD-TDP’ OR ‘HDDD1’ OR ‘HDDD2’ OR ‘tvFTD’ OR ‘FLDEM’ OR ‘semantic dementia’ OR ‘primary progressive aphasia’ OR ‘Mesulams Syndrome’ OR ‘Mesulam Syndrome’ OR C9orF72 OR ‘Corticobasal degeneration’ OR ‘primary supranuclear palsy’ OR ‘Demencia Frontotemporal’ OR ‘Degeneracion Lobar frontotemporal’ OR ‘Enfermedad de Pick’ OR ‘Complejo de Pick’ OR ‘Complejo Parkinson-Demencia’ OR ‘Demencia Semantica’ OR ‘afasia progresiva primaria’ OR ‘Paralisis supranuclear progresiva’ OR ‘syndrome cortico basal’) AND ('Argentina'/exp OR 'Bolivia'/exp OR 'Brazil'/exp OR 'Chile'/exp OR 'Colombia'/exp OR 'Costa Rica'/exp OR 'Cuba'/exp OR 'Dominican Republic'/exp OR 'Ecuador'/exp OR 'French Guiana'/exp OR 'Guatemala'/exp OR 'Haiti'/exp OR 'Honduras'/exp OR 'Mexico'/exp OR 'Nicaragua'/exp OR 'Panama'/exp OR 'Paraguay'/exp OR 'Peru'/exp OR 'Puerto Rico'/exp OR 'Uruguay'/exp OR 'Venezuela'/exp OR ‘Latin America*’:ti,ab,kw OR Argentin*:ti,ab,kw OR Bolivia*:ti,ab,kw OR Brazil:ti,ab,kw OR Brasil:ti,ab,kw OR Brazilian*:ti,ab,kw OR Chile:ti,ab,kw OR Chilean*:ti,ab,kw OR Colombia*:ti,ab,kw OR ‘Costa Rica*’:ti,ab,kw OR Cuba:ti,ab,kw OR Cuban*:ti,ab,kw OR Dominican*:ti,ab,kw OR Quisqueyan*:ti,ab,kw OR Ecuador*:ti,ab,kw OR ‘French Guiana’:ti,ab,kw OR ‘French Guianese’:ti,ab,kw OR ‘Guayana Francesa’:ti,ab,kw OR Guianan*:ti,ab,kw OR Guatemala*:ti,ab,kw OR Haiti*:ti,ab,kw OR Honduras:ti,ab,kw OR Honduran*:ti,ab,kw OR Mexico:ti,ab,kw OR Mexican*:ti,ab,kw OR Nicaragua*:ti,ab,kw OR Panama*:ti,ab,kw OR Paraguay*:ti,ab,kw OR Guaraní:ti,ab,kw OR Peru:ti,ab,kw OR Peruvian*:ti,ab,kw OR ‘Puerto Rico’:ti,ab,kw OR ‘Puerto Rican*’:ti,ab,kw OR Uruguay*:ti,ab,kw OR Venezuela*:ti,ab,kw OR Hispanic*:ti,ab,kw OR Latino*:ti,ab,kw OR Latina*:ti,ab,kw)

**Ovid-Medline All**

=65 results on 9/14/2020

(exp Frontotemporal Dementia/ OR exp Aphasia, Primary Progressive/ OR ("frontotemporal dementia" OR "frontotemporal lobe dementia" OR "frontotemporal lobe dementia" OR "frontal dementia" OR "frontal lobe dementia" OR "Wilhelmsen Lynch disease" OR "frontotemporal lobar degeneration" OR "disinhibition dementia Parkinsonism amyotrophy complex" OR "familial Picks disease" OR "Pick complex" OR "Picks complex" OR "hereditary dysphasic disinhibition dementia" OR "FTDP-17" OR DDPAC OR "FTD-GRN" OR "FTD-PGRN" OR "FTLD-17 GRN" OR "FTLD-TDP" OR HDDD1 OR HDDD2 OR tvFTD OR FLDEM OR "semantic dementia" OR "primary progressive aphasia" OR "Mesulams Syndrome" OR "Mesulam Syndrome" OR C9orF72 OR "Corticobasal degeneration" OR "primary supranuclear palsy" OR "Demencia Frontotemporal" OR "Degeneracion Lobar frontotemporal" OR "Enfermedad de Pick" OR "Complejo de Pick" OR "Complejo Parkinson Demencia" OR "Demencia Semantica" OR "afasia progresiva primaria" OR "Paralisis supranuclear progresiva" OR "syndrome cortico basal").ti,ab.) AND (Latin America/ OR Argentina/ OR Bolivia/ OR Brazil/ OR Chile/ OR Colombia/ OR Costa Rica/ OR Cuba/ OR Dominican Republic/ OR Ecuador/ OR El Salvador/ OR French Guiana/ OR Guatemala/ OR Haiti/ OR Honduras/ OR Mexico/ OR Nicaragua/ OR Panama/ OR Paraguay/ OR Peru/ OR Puerto Rico/ OR Uruguay/ OR Venezuela/ OR exp Hispanic Americans/ OR ("Latin America*" OR Argentin* OR Bolivia* OR Brazil OR Brasil OR Brazilian* OR Chile OR Chilean* OR Colombia* OR "Costa Rica*" OR Cuba OR Cuban* OR Dominican* OR Quisqueyan* OR Ecuador* OR "French Guiana" OR "French Guianese" OR "Guayana Francesa" OR Guianan* OR Guatemala* OR Haiti* OR Honduras OR Honduran* OR Mexico OR Mexican* OR Nicaragua* OR Panama* OR Paraguay* OR Guarani OR Peru OR Peruvian* OR "Puerto Rico" OR "Puerto Rican*" OR Uruguay* OR Venezuela* OR Hispanic* OR Latino* OR Latina*).ti,ab.)

**PsycInfo**

=51 results on 9/14/2020

(DE "Semantic Dementia" OR "frontotemporal dementia" OR "frontotemporal lobe dementia" OR "frontotemporal lobe dementia" OR "frontal dementia" OR "frontal lobe dementia" OR "Wilhelmsen Lynch disease" OR "frontotemporal lobar degeneration" OR "disinhibition dementia Parkinsonism amyotrophy complex" OR "familial Picks disease" OR "Pick complex" OR "Picks complex" OR "hereditary dysphasic disinhibition dementia" OR "FTDP-17" OR DDPAC OR "FTD-GRN" OR "FTD-PGRN" OR "FTLD-17 GRN" OR "FTLD-TDP" OR HDDD1 OR HDDD2 OR tvFTD OR FLDEM OR "semantic dementia" OR "primary progressive aphasia" OR "Mesulams Syndrome" OR "Mesulam Syndrome" OR C9orF72 OR "Corticobasal degeneration" OR "primary supranuclear palsy" OR "Demencia Frontotemporal" OR "Degeneracion Lobar frontotemporal" OR "Enfermedad de Pick" OR "Complejo de Pick" OR "Complejo Parkinson Demencia" OR "Demencia Semantica" OR "afasia progresiva primaria" OR "Paralisis supranuclear progresiva" OR "syndrome cortico basal") AND ("Latin America*" OR AB(Argentin*) OR TI(Argentin*) OR AB(Bolivia*) OR TI(Bolivia*) OR AB(Brazil) OR TI(Brazil) OR AB(Brasil) OR TI(Brasil) OR AB(Brazilian*) OR TI(Brazilian*) OR AB(Chile) OR TI(Chile) OR AB(Chilean*) OR TI(Chilean*) OR AB(Colombia*) OR TI(Colombia*) OR AB("Costa Rica*") OR TI(“Costa Rica”) OR AB(Cuba) OR TI(Cuba) OR AB(Cuban*) OR TI(Cuban*) OR AB(Dominican*) OR TI(Dominican*) OR AB(Quisqueyan*) OR TI(Quisqueyan*) OR AB(Ecuador*) OR TI(Ecuador*) OR AB("French Guiana") OR TI(“French Guiana”) OR AB("French Guianese") OR TI("French Guianese") OR AB("Guayana Francesa") OR TI("Guayana Francesa") OR AB(Guianan*) OR TI(Guianan*) OR AB(Guatemala*) OR TI(Guatemala*) OR AB(Haiti*) OR TI(Haiti*) OR AB(Honduras) OR TI(Honduras) OR AB(Honduran*) OR TI(Honduran*) OR AB(Mexico) OR TI(Mexico) OR AB(Mexican*) OR TI(Mexican*) OR AB(Nicaragua*) OR TI(Nicaragua*) OR AB(Panama*) OR TI(Panama*) OR AB(Paraguay*) OR TI(Paraguay*) OR AB(Guarani) OR TI(Guarani) OR AB(Peru) OR TI(Peru) OR AB(Peruvian*) OR TI(Peruvian*) OR AB("Puerto Rico") OR TI(“Puerto Rico”) OR AB("Puerto Rican*") OR TI(“Puerto Rican*”) OR AB(Uruguay*) OR TI(Uruguay*) OR AB(Venezuela*) OR TI(Venezuela*) OR AB(Hispanic*) OR TI(Hispanic*) OR AB(Latino*) OR TI(Latino*) OR AB(Latina*) OR TI(Latina*))

**Scopus**

=116 results on 9/14/2020

TITLE-ABS-KEY(“frontotemporal dementia” OR “frontotemporal lobe dementia” OR “frontotemporal lobe dementia” OR “frontal dementia” OR “frontal lobe dementia” OR “Wilhelmsen Lynch disease” OR “frontotemporal lobar degeneration” OR “disinhibition dementia Parkinsonism amyotrophy complex” OR “familial Picks disease” OR “Pick complex” OR “Picks complex” OR “hereditary dysphasic disinhibition dementia” OR “FTDP-17” OR “DDPAC” OR “FTD-GRN” OR “FTD-PGRN” OR “FTLD-17 GRN” OR “FTLD-TDP” OR “HDDD1” OR “HDDD2” OR “tvFTD” OR “FLDEM” OR “semantic dementia” OR “primary progressive aphasia” OR “Mesulams Syndrome” OR “Mesulam Syndrome” OR C9orF72 OR “Corticobasal degeneration” OR “primary supranuclear palsy” OR “Demencia Frontotemporal” OR “Degeneracion Lobar frontotemporal” OR “Enfermedad de Pick” OR “Complejo de Pick” OR “Complejo Parkinson-Demencia” OR “Demencia Semantica” OR “afasia progresiva primaria” OR “Paralisis supranuclear progresiva” OR “syndrome cortico basal”) AND TITLE-ABS-KEY(“Latin America*” OR Argentin* OR Bolivia* OR Brazil OR Brasil OR Brazilian* OR Chile OR Chilean* OR Colombia* OR “Costa Rica*” OR Cuba OR Cuban* OR Dominican* OR Quisqueyan* OR Ecuador* OR “French Guiana” OR “French Guianese” OR “Guayana Francesa” OR Guianan* OR Guatemala* OR Haiti* OR Honduras OR Honduran* OR Mexico OR Mexican* OR Nicaragua* OR Panama* OR Paraguay* OR Guaraní OR Peru OR Peruvian* OR “Puerto Rico” OR “Puerto Rican*” OR Uruguay* OR Venezuela* OR Hispanic* OR Latino* OR Latina*)

**Cochrane Library**

= 4 results (all 4 results are systematic reviews) on 9/14/2020

ID Search Hits

#1 MeSH descriptor: [Frontotemporal Dementia] explode all trees 55

#2 MeSH descriptor: [Aphasia, Primary Progressive] explode all trees 47

#3 ("frontotemporal dementia" OR "frontotemporal lobe dementia" OR "frontotemporal lobe dementia" OR "frontal dementia" OR "frontal lobe dementia" OR "Wilhelmsen Lynch disease" OR "frontotemporal lobar degeneration" OR "disinhibition dementia Parkinsonism amyotrophy complex" OR "familial Picks disease" OR "Pick complex" OR "Picks complex" OR "hereditary dysphasic disinhibition dementia" OR "FTDP-17" OR DDPAC OR "FTD-GRN" OR "FTD-PGRN" OR "FTLD-17 GRN" OR "FTLD-TDP" OR HDDD1 OR HDDD2 OR tvFTD OR FLDEM OR "semantic dementia" OR "primary progressive aphasia" OR "Mesulams Syndrome" OR "Mesulam Syndrome" OR C9orF72 OR "Corticobasal degeneration" OR "primary supranuclear palsy" OR "Demencia Frontotemporal" OR "Degeneracion Lobar frontotemporal" OR "Enfermedad de Pick" OR "Complejo de Pick" OR "Complejo Parkinson Demencia" OR "Demencia Semantica" OR "afasia progresiva primaria" OR "Paralisis supranuclear progresiva" OR "syndrome cortico basal") 402

#4 #1 OR #2 OR #3 404

#5 (("Latin America*" OR Argentin* OR Bolivia* OR Brazil OR Brasil OR Brazilian* OR Chile OR Chilean* OR Colombia* OR "Costa Rica*" OR Cuba OR Cuban* OR Dominican* OR Quisqueyan* OR Ecuador* OR "French Guiana" OR "French Guianese" OR "Guayana Francesa" OR Guianan* OR Guatemala* OR Haiti* OR Honduras OR Honduran* OR Mexico OR Mexican* OR Nicaragua* OR Panama* OR Paraguay* OR Guarani OR Peru OR Peruvian* OR "Puerto Rico" OR "Puerto Rican*" OR Uruguay* OR Venezuela* OR Hispanic* OR Latino* OR Latina*)):ti,ab,kw 19429

#6 #4 AND #5 4

**LILACS (Literatura Latino-Americana e do Caribe em Ciências da Saúde)**

=22 results on 9/14/2020

[Note: This search only worked in the Advanced Search builder at this link: <http://bases.bireme.br/cgi-bin/wxislind.exe/iah/online/?IsisScript=iah/iah.xis&base=LILACS&lang=p&form=A>]

(MH Demencia Frontotemporal OR MH Afasia Progresiva Primaria OR "frontotemporal dementia" OR "frontotemporal lobe dementia" OR "frontotemporal lobe dementia" OR "frontal dementia" OR "frontal lobe dementia" OR "Wilhelmsen Lynch disease" OR "frontotemporal lobar degeneration" OR "disinhibition dementia Parkinsonism amyotrophy complex" OR "familial Picks disease" OR "Pick complex" OR "Picks complex" OR "hereditary dysphasic disinhibition dementia" OR "FTDP-17" OR DDPAC OR "FTD-GRN" OR "FTD-PGRN" OR "FTLD-17 GRN" OR "FTLD-TDP" OR HDDD1 OR HDDD2 OR tvFTD OR FLDEM OR "semantic dementia" OR "primary progressive aphasia" OR "Mesulams Syndrome" OR "Mesulam Syndrome" OR C9orF72 OR "Corticobasal degeneration" OR "primary supranuclear palsy" OR "Demencia Frontotemporal" OR "Degeneracion Lobar frontotemporal" OR "Enfermedad de Pick" OR "Complejo de Pick" OR "Complejo Parkinson Demencia" OR "Demencia Semantica" OR "afasia progresiva primaria" OR "Paralisis supranuclear progresiva" OR "syndrome cortico basal") AND (MH América Latina OR MH Argentina OR MH Bolivia OR MH Brasil OR MH Chile OR MH Colombia OR MH Costa Rica OR MH Cuba OR MH Republica Dominicana OR MH Ecuador OR MH Guyana Francesa OR MH Guatemala OR MH Haiti OR MH Honduras OR MH Mexico OR MH Nicaragua OR MH Panama OR MH Paraguay OR MH Peru OR MH Puerto Rico OR MH Uruguay OR MH Venezuela OR MH Hispanoamericanos OR "Latin America" OR Argentin$ OR Bolivia$ OR Brazil OR Brasil OR Brazilian$ OR Chile OR Chilean$ OR Colombia$ OR "Costa Rica$" OR Cuba OR Cuban$ OR Dominican$ OR Quisqueyan$ OR Ecuador$ OR "French Guiana" OR "French Guianese" OR "Guayana Francesa" OR Guianan$ OR Guatemala$ OR Haiti$ OR Honduras OR Honduran$ OR Mexico OR Mexican$ OR Nicaragua$ OR Panama$ OR Paraguay$ OR Guarani OR Peru OR Peruvian$ OR "Puerto Rico" OR "Puerto Rican$" OR Uruguay$ OR Venezuela$ OR Hispanic$ OR Latino$ OR Latina$)

**SciElo.org**

=40 results on 9/14/2020

All Indexes: ("frontotemporal dementia" OR "primary progressive aphasia" OR "semantic dementia") AND ("Latin America*" OR Argentin* OR Bolivia* OR Brazil OR Brasil OR Brazilian* OR Chile OR Chilean* OR Colombia* OR "Costa Rica*" OR Cuba OR Cuban* OR Dominican* OR Quisqueyan* OR Ecuador* OR "French Guiana" OR "French Guianese" OR "Guayana Francesa" OR Guianan* OR Guatemala* OR Haiti* OR Honduras OR Honduran* OR Mexico OR Mexican* OR Nicaragua* OR Panama* OR Paraguay* OR Guarani OR Peru OR Peruvian* OR "Puerto Rico" OR "Puerto Rican*" OR Uruguay* OR Venezuela* OR Hispanic* OR Latino* OR Latina*)
